# Efficacy and Safety of Combination Therapy with PARP Inhibitors and Anti-Angiogenic Agents in Ovarian Cancer: A Systematic Review and Meta-Analysis

**DOI:** 10.3390/jcm14051776

**Published:** 2025-03-06

**Authors:** István Baradács, Brigitta Teutsch, Ádám Vincze, Péter Hegyi, Bence Szabó, Péter Nyirády, Nándor Ács, Zsolt Melczer, Ferenc Bánhidy, Balázs Lintner

**Affiliations:** 1 Centre for Translational Medicine, Semmelweis University, 1085 Budapest, Hungarylintnerster@gmail.com (B.L.); 2 Department of Obstetrics & Gynecology, Semmelweis University, 1082 Budapest, Hungary; 3 Institute for Translational Medicine, Medical School, University of Pécs, 7624 Pecs, Hungary; 4 Department of Radiology, Medical Imaging Centre, Semmelweis University, 1082 Budapest, Hungary; 5 Institute of Pancreatic Diseases, Semmelweis University, 1083 Budapest, Hungary; 6 Department of Urology, Semmelweis University, 1082 Budapest, Hungary

**Keywords:** ovarian cancer, PARP inhibitor, anti-angiogenic agent, combination therapy, progression-free survival

## Abstract

**Introduction:** Ovarian cancer is a significant contributor to gynecological cancer-related mortality, necessitating innovative treatment strategies. This systematic review and meta-analysis aimed to assess the efficacy and safety of combining PARP inhibitors with anti-angiogenic agents (AAAs) in the treatment of ovarian cancer. **Methods:** This study adhered to the Preferred Reporting Items for Systematic Reviews and Meta-Analysis (PRISMA) guidelines and was registered on PROSPERO (CRD42022319461). A systematic search of three electronic databases, including MEDLINE (via PubMed), EMBASE, and Cochrane Library was conducted to identify relevant randomized controlled trials (RCT) that evaluated the efficacy and safety of the combination therapy. Subgroup analyses were based on BRCA mutation status. Meta-analysis was conducted to estimate pooled hazard ratios (HR) and risk ratios (RR) for progression-free survival (PFS) and adverse events, respectively. The combination therapy was compared to PARP inhibitors alone and to chemotherapy. Heterogeneity was assessed using Higgins and Thompson’s I^2^ statistic where applicable. **Results:** Seven RCTs involving 2397 patients were included. Combination therapy did not show a statistically significant improvement in PFS compared to PARP inhibitor monotherapy in the general population (HR 0.63, CI 0.37–1.06), or in BRCA-mutated (HR 0.70, CI 0.30–1.63) and BRCA wild-type subgroups (HR 0.39, CI 0.14–1.07). When compared to chemotherapy, combination therapy produced no significant PFS benefit in recurrent ovarian cancer (HR 0.83, CI 0.42–1.63) in the total population. Safety analysis revealed that hypertension and diarrhea were significantly more frequent in combination therapy compared with PARP inhibitors alone (RR 6.80, CI 2.87–16.06 and RR 10.04, CI 2.25–44.75) or chemotherapy alone (RR 13.80, CI 3.43–55.57 and RR 6.57, CI 2.84–15.24). **Conclusions:** The combination of PARP inhibitors and AAAs did not demonstrate a statistically significant benefit in PFS compared to PARP inhibitors or chemotherapy alone in recurrent ovarian cancer. While the combination therapy was generally well tolerated, hypertension and diarrhea occurred significantly. These findings suggest that combination therapy may not provide a clear survival advantage in the recurrent setting. Further high-quality, biomarker-driven clinical trials are needed to refine patient selection, optimize toxicity management, and determine the potential role of combination therapy in ovarian cancer treatment.

## 1. Introduction

Ovarian cancer is the second leading cause of death from gynecologic cancers worldwide, with a five-year survival rate of less than 50% in advanced stages [1]. The majority of patients relapse after platinum-based therapy, with a median PFS of approximately 12–18 months in the first-line setting [2]. Conventional therapies, especially in the recurrent setting, often result in diminishing returns with increased toxicity and reduced efficacy over time [3]. This highlights the need for novel treatment strategies.

PARP inhibitors have gained attention in the treatment of ovarian cancer for their promising outcomes in recent years. PARP inhibitors have demonstrated significant effectiveness in patients with homologous recombination deficiency (HRD), particularly in those with BRCA1/2 mutations. Clinical trials have shown that these agents improve PFS by a range of 11.2 to 21 months, compared to only 5.5 months with a placebo [4]. Synthetic lethality targets tumor cells with impaired DNA repair mechanisms, causing DNA damage and tumor cell death [5]. PARP inhibitors have the ability to treat ovarian cancer even in patients without BRCA mutations; however, their effectiveness may vary [6].

Another crucial objective in the treatment of ovarian cancer is to target the vascularization of the tumor. Angiogenesis is an essential process for tumor growth and survival, as it provides tumor cells with the necessary blood supply [7]. AAAs such as bevacizumab target the VEGF pathway and are effective in solid tumors such as ovarian cancer [8]. These agents disrupt the tumor microenvironment by inhibiting angiogenesis [9].

The combination of PARP inhibitors with AAAs for ovarian cancer shows potential due to their synergistic mechanisms of action and beneficial effects. AAAs suppress VEGF-driven angiogenesis, preventing tumor adaptation and improving treatment response. By normalizing vasculature, they enhance blood perfusion, improving PARP inhibitor delivery and tumor penetration [10]. Hypoxia induced by antiangiogenic therapy downregulates homologous recombination repair proteins (BRCA1/2, RAD51), increasing tumor reliance on PARP-mediated repair, thereby amplifying synthetic lethality [11]. Additionally, this combination modifies the immune microenvironment, as PARP inhibitors increase tumor immunogenicity, while antiangiogenic agents counteract VEGF-driven immunosuppression, boosting anti-tumor immunity [12]. Despite the observed benefits of combination therapy, current guidelines such as those from the National Comprehensive Cancer Network (NCCN) and the European Society for Medical Oncology (ESMO) do not yet recommend this approach, favoring monotherapy with PARP inhibitors or chemotherapy instead [11,13].

This systematic review and meta-analysis aimed to evaluate the efficacy and safety of combining PARP inhibitors with AAAs in the treatment of ovarian cancer compared to PARP inhibitor monotherapy and chemotherapy.

## 2. Methods

The investigation was documented in accordance with the 2020 Statement of the Preferred Reporting Items for Systematic Reviews and Meta-Analysis (PRISMA), as indicated in Table S1 [14]. This study was conducted in adherence to the guidelines for Systematic Reviews of Intervention as outlined in the Cochrane Handbook [15]. The protocol for the review was registered on PROSPERO under registration number CRD42022319461.

### 2.1. Search Strategy

A systematic search was performed to identify relevant studies in electronic databases, including MEDLINE (via PubMed), EMBASE, and Cochrane Library from the inception of the databases until 12 February 2025. The search was conducted in duplicate by two independent authors (IB and ÁV). The search strategy utilized a combination of relevant keywords and Medical Subject Headings (MeSH) terms, including “ovarian cancer”, “PARP inhibitors”, “anti-angiogenic agents”, and “combination therapy” (see Appendix S1). Reference lists of eligible articles were screened manually to ensure the inclusion of all potentially relevant articles. No language or other constraints were utilized.

### 2.2. Selection, Eligibility Criteria, and Data Extraction

RCTs evaluating the efficacy and safety of PARP inhibitor and AAA combination treatment for ovarian cancer were included. Eligibility criteria for the study were as follows: (1) studied participants diagnosed with ovarian, fallopian tube, or peritoneal cancer (collectively defined as ovarian cancer); (2) examined both newly diagnosed and recurrent cases; (3) included patients with any number of prior therapies; (4) patients who had previously received AAAs, PARP inhibitors, or chemotherapy were eligible for inclusion; and (5) considered Phase I RCTs and conference papers as eligible sources of information. Trials investigating PARP inhibitors in combination with other targeted therapeutic medicines were not included.

After duplicate removal, two authors (IB and ÁV) independently selected articles by reviewing titles, abstracts, and full texts, while ensuring that they met the eligibility criteria. Cohen’s kappa coefficient was computed for each selection phase [16]. Differences were resolved by a third reviewer (BL). Two reviewers separately extracted data using a predetermined form (IB, ÁV). Study characteristics (author, publication year, and study design), patient characteristics (number of patients, age, and BRCA mutation status), intervention details (PARP inhibitor and anti-angiogenic combination therapy), outcome data (PFS and AEs), and risk ratios (RRs) or hazard ratios (HRs) with 95% CIs were extracted.

### 2.3. Risk of Bias and Quality of the Evidence Assessment

The studies included were independently examined by two authors (IB, and ÁV) using the Revised Cochrane risk-of-bias assessment for RCTs (RoB 2) [17]. This technique assesses bias in five areas: randomization method, variations from intended treatments, missing outcome data, outcome measurement, and result selection. Differences were addressed by a third reviewer (BL). We assessed evidence reliability using the Grading of Recommendations Assessment, Development, and Evaluation (GRADE) method [18]. Two independent reviewers (IB and ÁV) assessed each outcome and comparison criterion, and a third reviewer resolved disagreements (BL).

### 2.4. Synthesis Methods and Statistical Analysis

The purpose of this meta-analysis was to synthesize the data and estimate the combined effect of PARP inhibitor and combination therapy on clinical outcomes.

PFS was the primary outcome of interest. For such time-to-event data, hazard ratios (HR) with 95% confidence intervals (CI) served as the effect size measure. Pooled HRs were computed by first calculating the logarithm of HRs and their standard errors (SE), following the methodology outlined by Tierney et al. (2007) [19]. The inverse variance weighting method was applied on a logarithmic scale to estimate the pooled HR. The restricted maximum-likelihood estimator and the Q profile method were used for confidence intervals [20,21].

For the AE binary variables, risk ratios (RR) with 95% confidence intervals (CI) were used as the measure of effect size. To compute study-specific and pooled risk ratios, we extracted the total number of patients and the number of events of interest in each group (referred to as “raw data”) from studies where this information was available. The results indicate the risk of the event occurring in the experimental group compared to the control group. When zero-cell counts were present, individual study RRs with 95% CIs were calculated using a continuity correction of 0.5. The pooled RR was estimated using the inverse variance weighting method. Similar to HR calculations, for direct RR calculation, the restricted maximum-likelihood estimator was applied, with the Q profile method used to determine confidence intervals [20,21].

Statistical significance was determined either based on whether the pooled CI excluded the null effect value or by a preset alpha value of 0.05. The meta-analysis findings were summarized using forest plots and aggregated forest plots.

In settings with a minimum number of three RCTs, between-study heterogeneity was assessed using Higgins and Thompson’s I^2^ statistic [22]. The interpretation of I^2^ follows the Cochrane Collaboration Handbook guidelines: 0% to 40% indicates heterogeneity might not be important, 30% to 60% may represent moderate heterogeneity, 50% to 90% may indicate substantial heterogeneity and greater than 75% suggests considerable heterogeneity.

All statistical analyses were conducted in R [23] using the meta package [24].

## 3. Results

### 3.1. Search and Selection

**Figure 1:**
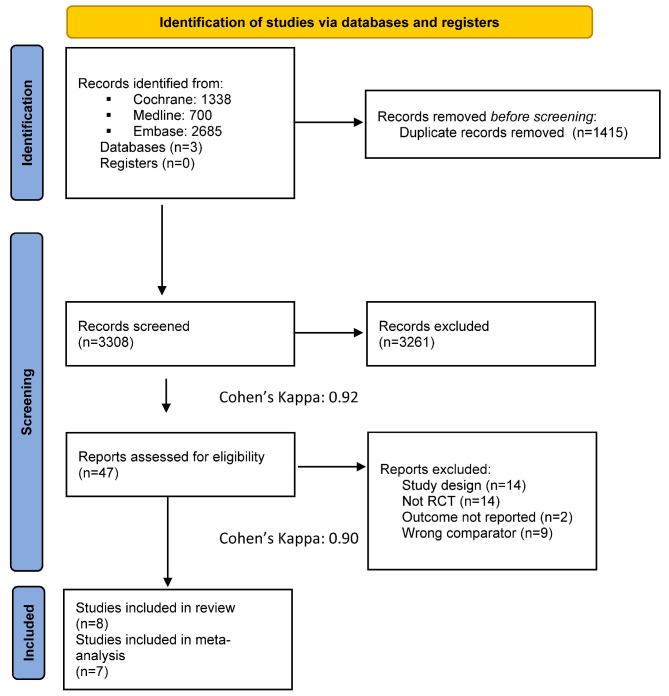
PRISMA 2020 flowchart representing the study selection process.

Our search methodology yielded a total of 4723 articles. Seven RCTs were deemed eligible based on the inclusion criteria and were subsequently selected for further analysis. Figure 1 shows the process of study selection.

### 3.2. Basic Characteristics of Studies Included

The studies encompassed a collective sample size of 2157 patients. The included trials varied in phase, with both phase II and phase III studies assessing the efficacy of combination therapy in recurrent or newly diagnosed ovarian cancer. The studies compared combination therapy with either PARP inhibitor monotherapy or chemotherapy, while variations in treatment regimens, dosing schedules, and patient selection criteria were observed across studies. A significant proportion of participants had prior exposure to platinum-based chemotherapy, and BRCA mutation status was a key stratification factor in several trials. Table 1 provides a summary of the main characteristics of the investigations.

### 3.3. Survival Analysis

#### 3.3.1. Recurrent Ovarian Cancer

##### Combination Therapy vs. PARP Inhibitors Alone

**Figure 2:**
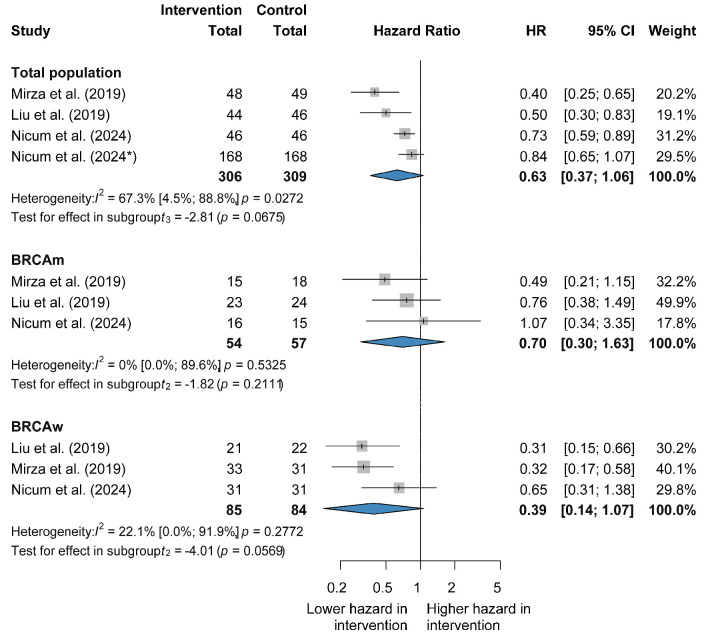
Forest plot representing the efficacy of combination therapy vs. PARP inhibitor alone in recurrent ovarian cancer. The asterisk (*) indicates that this is the second publication by the same author in the same year.

The findings of four RCTs indicate that the combination did not show a statistically significant improvement in PFS compared to PARP inhibitor therapy in the general population (HR 0.63, CI 0.37–1.06). Substantial heterogeneity was detected (I^2^ = 67.3%, *p* = 0.0272), which is statistically significant. Combination therapy has not demonstrated a statistically significant benefit in patients with BRCA mutations (HR 0.70, CI 0.30–1.63) and no heterogeneity was detected (I^2^ = 0%, *p* = 0.5325, not statistically significant). Similarly, no statistically significant PFS advantage was observed in the BRCA wild-type population (HR 0.39, CI 0.14–1.07) with low heterogeneity (I^2^ = 22.1%, *p* = 0.2772, not statistically significant) [26,27,28,33] (Figure 2 and Figure S1).

##### Combination Therapy vs. Chemotherapy Alone

**Figure 3:**
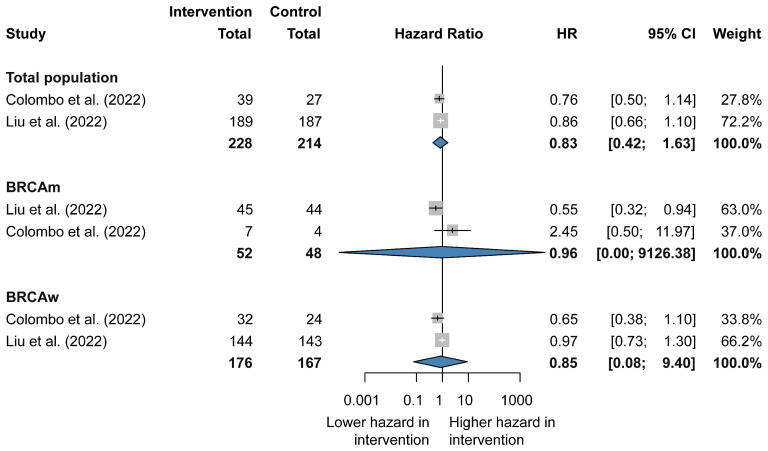
Forest plot representing the efficacy of combination therapy vs. chemotherapy alone in recurrent ovarian cancer.

Two clinical trials were analyzed to evaluate the efficacy of combination therapy compared with chemotherapy. The results showed that no significant PFS benefit was observed in the total population (HR 0.83, CI 0.42–1.63), as well as in the BRCA-mutated (HR 0.96, CI 0.00–9126.38) and BRCA wild-type (HR 0.85, CI 0.08–9.40) subgroups [25,29,30] (Figure 3 and Figure S2).

#### 3.3.2. Newly Diagnosed Ovarian Cancer

##### Combination Therapy vs. AAA Alone

In this setting, one trial [10,32] demonstrated that combination therapy significantly improved PFS in the total population (HR 0.59, CI 0.49–0.72) and the BRCA-mutated (HR 0.31 CI 0.20–0.47) and BRCA wild-type groups (HR 0.63 CI 0.51–0.77).

### 3.4. Safety in Recurrent Ovarian Cancer

#### 3.4.1. Combination Therapy vs. PARP Inhibitor Alone

**Figure 4:**
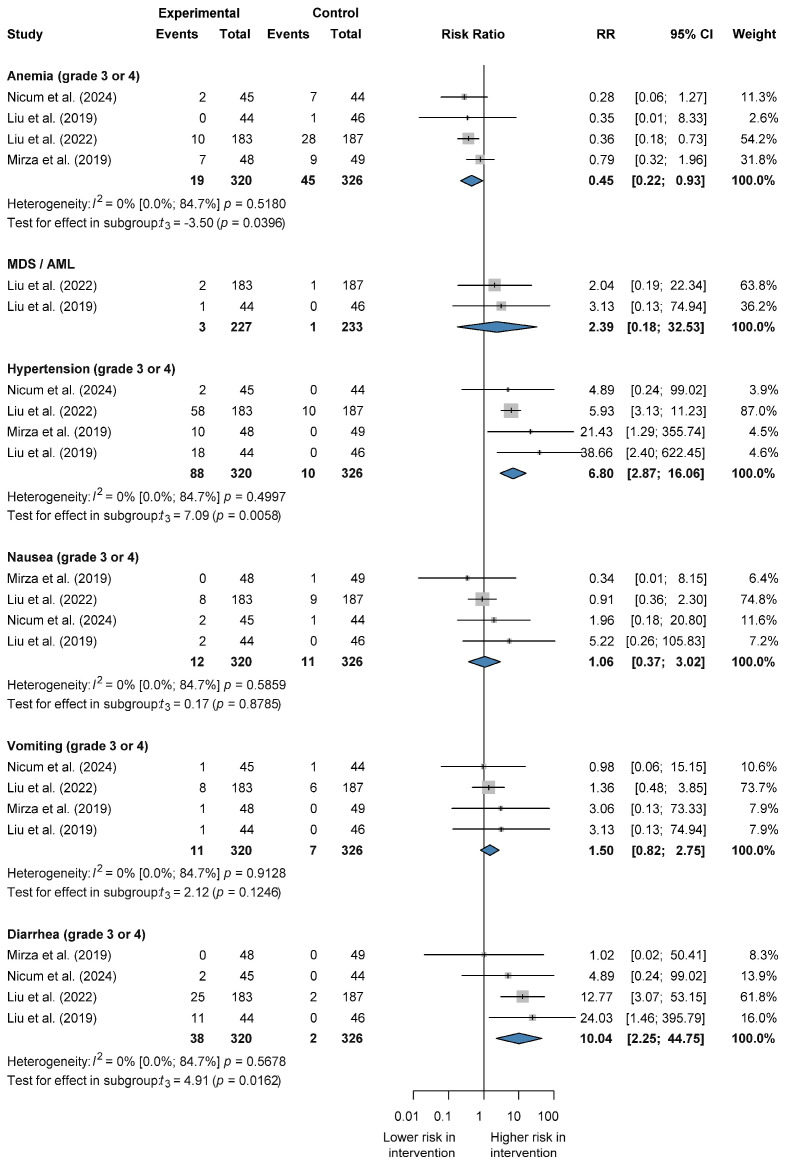
Forest plot representing risk ratios of grade 3 ≤ adverse events for combination therapy vs. PARP inhibitors alone in recurrent ovarian cancer.

There was no significant difference in the risk of severe grade 3, and 4 anemia (RR 0.45 CI 0.22–0.93); nausea (RR 1.06 CI 0.37–3.02); and vomiting (RR 1.50 CI 0.82–2.75) between combination therapy and PARP inhibitors. However, the risks of severe hypertension (RR 6.80 CI 2.87–16.06) and diarrhea (RR 10.04 CI 2.25–44.75) were significantly increased with combination therapy. No significant difference was observed in the risk of myelodysplastic syndrome/acute myeloid leukemia (MDS/AML) (RR 2.39 CI 0.18–32.53) compared with PARP inhibitor treatment. No heterogeneity was observed for anemia, hypertension, nausea, vomiting, or diarrhea (I^2^ = 0%, *p* not statistically significant) [26,27,28,31] (Figure 4 and Figure S3).

#### 3.4.2. Combination Therapy vs. Chemotherapy Alone

**Figure 5:**
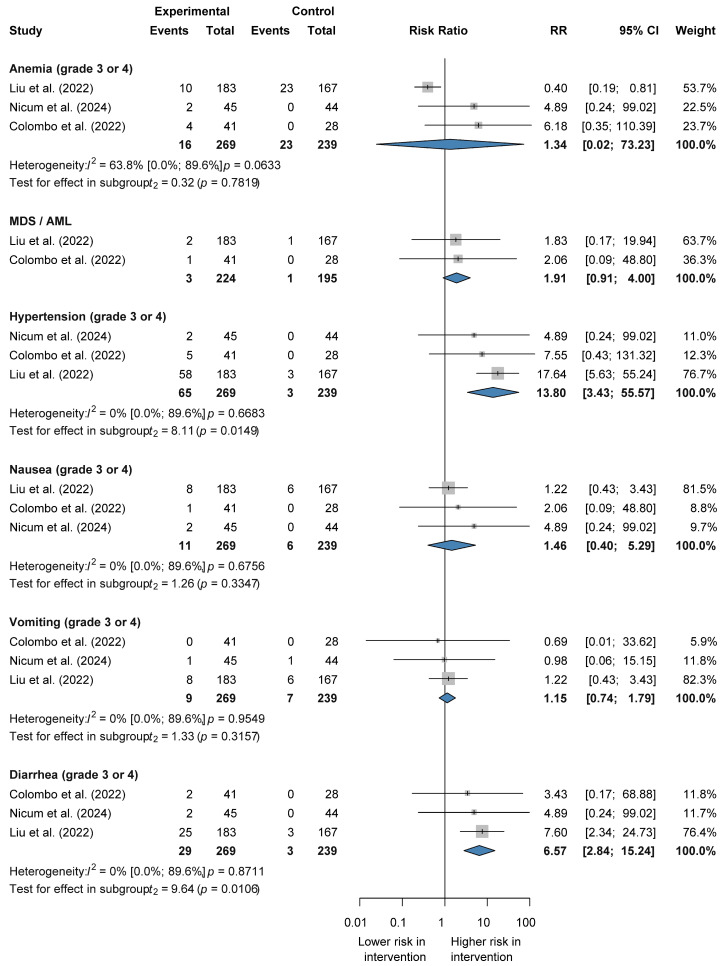
Forest plot representing risk ratios of grade 3 ≤ adverse events for combination therapy vs. chemotherapy alone in recurrent ovarian cancer.

In this therapeutic context, the risks of severe hypertension (RR 13.80 CI 3.43–55.57) and diarrhea (RR 6.57 CI 2.84–15.24) were significantly increased compared to chemotherapy alone. The combination therapy did not significantly escalate the risk of serious anemia (RR 1.34 CI 0.02–73.23), nausea (RR 1.46 CI 0.40–5.29), vomiting (RR 1.15 CI 0.74–1.79), and MDS/AML (RR 1.91 CI 0.91–4.00) in comparison to chemotherapy. Substantial heterogeneity was detected for anemia (I^2^ = 63.8%, *p* = 0.0633, not statistically significant), while no heterogeneity was observed for hypertension, nausea, vomiting, or diarrhea (I^2^ = 0%, *p* not statistically significant) [25,29,30,31] (Figure 5 and Figure S4).

### 3.5. Risk of Bias Assessment

The results of the assessment of the risk of bias are presented in Figure S5. It is important to note that five of the analyzed studies were conducted in an open-label manner, and one case relied on data from a conference abstract. As a result, the overall risk of bias remains unclear.

### 3.6. Quality of Evidence

In general, the certainty of evidence is low, primarily due to inconsistencies in the pooled results. In three cases of serious AEs—hypertension and diarrhea in combination therapy vs. PARP inhibitor alone and diarrhea in combination therapy vs. chemotherapy alone—the certainty of evidence was moderate. A detailed description of evidence quality can be found in Supplementary Tables S2–S5.

## 4. Discussion

Because of their complementary mechanisms of action, combination therapy with PARP inhibitors and AAAs has demonstrated promising efficacy in the treatment of ovarian cancer. In terms of PFS in the general population, our meta-analysis revealed that combination therapy did not show a statistically significant improvement compared to PARP inhibitors or chemotherapy alone. In patients with BRCA mutations, no statistically significant PFS benefit was observed. As well, in the BRCA wild-type subgroup, no statistically significant improvement in PFS was detected. These results indicate that while combination therapy has been explored as an alternative treatment strategy, its superiority over monotherapy in the recurrent setting has not been established [10,27].

The differential response to combination therapy, affected by genetic factors such as BRCA mutations, emphasizes the value of genetic profiling in ovarian cancer. Although our results did not confirm a statistically significant difference in PFS based on BRCA mutation status, the numerical trends suggest a potential variation in response between BRCA-mutated and wild-type populations. In trials such as the AVANOVA2 [28] and NRG-GY004 [27] studies, differences in outcomes between BRCA-mutated and BRCA wild-type patients have been reported, highlighting the potential for genetic factors to influence treatment efficacy. However, given the lack of statistical significance in our pooled results, further research is necessary to determine whether genetic profiling can be reliably used to guide the selection of combination therapy.

Angiogenesis is essential for tumor growth and survival, and anti-angiogenic compounds have been shown to be effective against ovarian cancer and other solid tumors. Preclinical studies imply that combining PARP inhibitors with AAAs may enhance the DNA damage response and PARP inhibition [9]. The synergistic effect of combination therapy may be attributed to the down-regulation of genes involved in the homologous recombination system by cediranib. This down-regulation potentially enhances the effectiveness of Olaparib, indicating a significant interaction between these two agents in the treatment of ovarian cancer [34]. Despite these mechanistic advantages, our findings indicate that the combination therapy did not produce a significant improvement in PFS when compared to chemotherapy alone in the total population. Moreover, no statistically significant PFS advantage was observed in the BRCA-mutated or BRCA wild-type subgroups compared to chemotherapy alone. This suggests that in the recurrent setting, combination therapy may not provide a clear survival advantage over standard chemotherapy.

In the context of newly diagnosed ovarian cancer, the PAOLA-1 trial [10] demonstrated that combining Olaparib and bevacizumab significantly improved PFS in patients, regardless of their BRCA mutation status. Among patients with HRD-positive tumors, including those without BRCA mutations, this benefit was particularly striking. These findings suggest that the combination of PARP inhibitors and AAAs can be beneficial for a wider range of patients in a first-line setting.

The safety profile of combination therapy remains a crucial consideration in treatment decision-making. The risks of MDS/AML, serious anemia, nausea, and vomiting were not significantly different between combination therapy and either PARP inhibitors or chemotherapy, whereas hypertension and diarrhea were notably more frequent with combination therapy. Hypertension is a well-documented side effect of VEGF inhibitors due to the VEGF blockade, which reduces nitric oxide production, leading to increased vascular resistance [35]. Additionally, PARP inhibitors may contribute to vascular dysfunction by affecting oxidative stress, potentially amplifying this effect [36]. Proactive blood pressure monitoring and antihypertensive strategies are essential for patients undergoing treatment. Diarrhea possibly results from the combined impact of VEGF inhibition, which compromises intestinal microvascular integrity, and PARP inhibition, which affects rapidly proliferating epithelial cells [37,38]. Supportive care, including hydration and dietary modifications, is crucial for symptom management.

Given these findings, individualized toxicity management is essential, particularly for patients at higher risk of cardiovascular or gastrointestinal complications. Future studies should explore biomarkers to predict toxicity risk, allowing for a more personalized approach to combination therapy.

### 4.1. Heterogeneity

Heterogeneity was substantial and statistically significant only for PFS in the overall population in the PARP comparison. In the chemotherapy comparison, heterogeneity was substantial for severe anemia but did not reach statistical significance. In all other settings, heterogeneity was low and not statistically significant, suggesting that observed differences may be due to chance. The substantial heterogeneity may stem from differences in patient populations, prior treatments, follow-up durations, study methodologies, and differences in baseline prognostic factors, such as HRD status or prior bevacizumab use, which could further contribute to variability. The lack of statistically significant heterogeneity in most comparisons suggests that the number of included trials is too small to detect true heterogeneity.

### 4.2. Implications for Practice and Research

The synthesis of the existing literature allows for the application of scientific findings to everyday activities, which is the highest priority [39]. The combination did not show a statistically significant PFS advantage in the recurrent setting. However, its efficacy in newly diagnosed ovarian cancer suggests potential benefits with early intervention.

Clinically, patient selection is critical, as response trends vary by BRCA status. The increased risk of hypertension and diarrhea necessitates proactive management, including early blood pressure control and gastrointestinal support. Future research should focus on biomarker-driven patient stratification and optimizing treatment sequencing to enhance efficacy and minimize resistance. Further studies should assess the impact of prior AAA exposure on combination therapy outcomes.

While combination therapy remains a potential option, its integration into practice should be guided by refined biomarker-driven trials to maximize benefits and reduce toxicity risks.

### 4.3. Strengths and Limitations

The strengths of this study include a comprehensive analysis of RCTs, allowing for a detailed evaluation of both efficacy and safety outcomes. The inclusion of subgroup analyses based on BRCA mutation status enhances the applicability of our findings to clinical practice. However, certain limitations must be acknowledged. The overall certainty of evidence is low due to inconsistencies in the pooled results, and five of the included studies were conducted in an open-label manner, which introduces a risk of bias. Additionally, one study was included based on conference abstract data, limiting the availability of full methodological details. The variability in trial design, drug regimens, and control arms further complicates direct comparisons across studies.

## 5. Conclusions

In conclusion, combination therapy with PARP inhibitors and AAAs did not demonstrate a statistically significant benefit in the investigated populations when compared to PARP inhibitors or chemotherapy alone. The safety analysis revealed that while the combination was generally well tolerated, serious hypertension and diarrhea were significantly more frequent compared to both PARP inhibitor monotherapy and chemotherapy. Further high-quality, randomized controlled trials with standardized protocols are required to confirm these findings and optimize treatment selection for patients with ovarian cancer.

## Figures and Tables

**Table 1:** Basic characteristics of studies included.

No.	Article(s)	Year	Trial	Phase	Study Design	Sample Size	Disease State (Newly Diagnosed/Recurrent)	Intervention Arm	Control Arm
1.	Liu et al. (2022) [25]	2022	NRG-GY004	III	randomized, open-label	565	Recurrent	Olaparib 200 mg orally twice a day with cediranib maleate 30 mg once a day/Olaparib 300 mg orally twice a day	PBC
2.	Liu et al. (2014, 2019) [26,27]	2014	NCT01116648	II	randomized, open-label	90	Recurrent	Olaparib 200 mg orally twice a day with cediranib 30 mg once a day	Olaparib 400 mg twice a day
3.	Mirza et al. (2019) [28]	2019	AVANOVA2	II	randomized, open-label	97	Recurrent	Niraparib 300 mg orally once a day with intravenous bevacizumab 15 mg/kg once every 3 weeks until disease progression	Niraparib 300 mg orally once a day
4.	Colombo et al. (2022) [29]	2022	BAROCCO	II	randomized, open-label	123	Recurrent	Olaparib 300 mg tablets twice a day together with 20 mg cediranib once a day (continuous schedule) or 5 days/week (intermittent schedule)	Paclitaxel, IV weekly, 80 mg/m^2^ up to 24 weeks
5.	Nicum et al. (2021, 2024) [30,31]	2024	OCTOVA	II	randomized, open-label	139	Recurrent	Olaparib, oral, 300 mg twice a day or with cediranib 20 mg orally once a day	Paclitaxel, IV weekly, 80 mg/m^2^
6.	Ray-Coquard et al. (2019) [10], Lorusso et al. (2024) [32]	2024	PAOLA-1	III	randomized, double-blind	806	Newly diagnosed	Olaparib 300 mg orally twice a day	Placebo
7.	Nicum et al. (2024/2) [33]	2024	ICON9	III	randomized	337	Recurrent	Olaparib, oral, 300 mg twice a day with cediranib 20 mg orally once a day	Olaparib, oral, 300 mg twice a day

PBC—platinum-based chemotherapy.

## Data Availability

All data generated or analyzed during the investigation are included in the published article and the Supplementary Materials.

## Supplementary Materials

The following supporting information can be downloaded at: https://www.mdpi.com/article/10.3390/jcm14051776/s1. Table S1: PRISMA 2020 checklist. Table S2: Summary of findings & quality of evidence—PFS in recurrent OC: combination therapy vs. PARP inhibitor alone. Table S3: Summary of findings & quality of evidence—PFS in recurrent OC: combination therapy vs. chemotherapy alone. Table S4: Summary of findings & quality of evidence—AEs in recurrent OC: combination therapy vs. PARP inhibitor alone. Table S5: Summary of findings & quality of evidence—AEs in recurrent OC: combination therapy vs. chemotherapy alone. Appendix S1. The search terms applied in a systematic search. Figure S1: Forest plot demonstrates that the combination therapy for recurrent ovarian cancer is non-inferior to PARP inhibitor alone in reducing the hazard ratio for disease progression or death in the analyzed populations. Figure S2: Forest plot demonstrates that the combination therapy for recurrent ovarian cancer is equally effective as chemotherapy alone in reducing the hazard ratio for disease progression or death in the analyzed populations. Figure S3: Forest plot demonstrates the impact of combination therapy versus PARP inhibitor alone on the risk of adverse events in patients with recurrent ovarian cancer. Figure S4: Forest plot demonstrates the impact of combination therapy versus chemotherapy alone on the risk of adverse events in patients with recurrent ovarian cancer. Figure S5: Risk of bias summary at study level: for each included trial.
