# Supplementary material for: Efficacy and Safety of Combination Therapy with PARP Inhibitors and Anti-Angiogenic Agents in Ovarian Cancer: A Systematic Review and Meta-Analysis
Source: J Clin Med. 2025 Mar 6;14(5):1776. doi: 10.3390/jcm14051776 (PMC11901299; doi:10.3390/jcm14051776)
Supplement: Supplementary file 1 [file jcm-14-01776-s001.zip › jcm-3474034-supplementary.pdf]

Efficacy and Safety of Combination Therapy with PARP Inhibitors and Anti-Angiogenic Agents in Ovarian Cancer: A Systematic Review and Meta-Analysis

Authors

István Baradács[1,2], Brigitta Teutsch[1,3,6], Ádám Vincze[1,2], Péter Hegyi[1,3,4], Andrea Harnos[1,3]

Péter Nyirády[1,5], Nándor Ács[1,2], Ferenc Bánhidy[1,2], Balázs Lintner[1,2]

Affiliations:

1. Centre for Translational Medicine, Semmelweis University, Budapest, Hungary
2. Department of Obstetrics & Gynecology, Semmelweis University, Budapest
3. Institute for Translational Medicine, Medical School, University of Pécs, Pécs, Hungary
4. Institute of Pancreatic Diseases, Semmelweis University, Budapest, Hungary
5. Department of Urology, Semmelweis University, Budapest, Hungary
6. Department of Radiology, Medical Imaging Centre, Semmelweis University, Budapest, Hungary

Corresponding author

Balázs Lintner MD, PhD

Department of Obstetrics and Gynecology

Semmelweis University

Postal address: H-1082 Budapest, Üllői út 78/A, Hungary

Tel.: +36 20 919 3312

E-mail address: lintnerster@gmail.com

Tables

Table S1 PRISMA 2020 checklist
Table S2 Summary of findings & quality of evidence – PFS in recurrent OC: combination therapy vs. PARP inhibitor alone
Table S3 Summary of findings & quality of evidence – PFS in recurrent OC: combination therapy vs. chemotherapy alone
Table S4 Summary of findings & quality of evidence – AEs in recurrent OC: combination therapy vs. PARP inhibitor alone
Table S5 Summary of findings & quality of evidence – AEs in recurrent OC: combination therapy vs. chemotherapy alone

Appendix

Appendix S1 The search terms applied in a systematic search

Figure legends

Figure S1 Forest plot demonstrates that the combination therapy for recurrent ovarian cancer is non-inferior to PARP inhibitor alone in reducing the hazard ratio for disease progression or death in the analyzed populations
Figure S2 Forest plot demonstrates that the combination therapy for recurrent ovarian cancer is equally effective as chemotherapy alone in reducing the hazard ratio for disease progression or death in the analyzed populations
Figure S3 Forest plot demonstrates the impact of combination therapy versus PARP inhibitor alone on the risk of adverse events in patients with recurrent ovarian cancer
Figure S4 Forest plot demonstrates the impact of combination therapy versus chemotherapy alone on the risk of adverse events in patients with recurrent ovarian cancer
Figure S5 Risk of bias summary at study level: for each included trial

Table S1 PRISMA 2020 checklist

Section and Topic	Item #	Checklist item	Location where item is reported
**TITLE**			
Title	1	Identify the report as a systematic review.	1
**ABSTRACT**			
Abstract	2	See the PRISMA 2020 for Abstracts checklist.	3
**INTRODUCTION**			
Rationale	3	Describe the rationale for the review in the context of existing knowledge.	4
Objectives	4	Provide an explicit statement of the objective(s) or question(s) the review addresses.	4
**METHODS**			
Eligibility criteria	5	Specify the inclusion and exclusion criteria for the review and how studies were grouped for the syntheses.	5
Information sources	6	Specify all databases, registers, websites, organisations, reference lists and other sources searched or consulted to identify studies. Specify the date when each source was last searched or consulted.	5
Search strategy	7	Present the full search strategies for all databases, registers and websites, including any filters and limits used.	7<br>Supplementary material
Selection process	8	Specify the methods used to decide whether a study met the inclusion criteria of the review, including how many reviewers screened each record and each report retrieved, whether they worked independently, and if applicable, details of automation tools used in the process.	5
Data collection process	9	Specify the methods used to collect data from reports, including how many reviewers collected data from each report, whether they worked independently, any processes for obtaining or confirming data from study investigators, and if applicable, details of automation tools used in the process.	5
Data items	10a	List and define all outcomes for which data were sought. Specify whether all results that were compatible with each outcome domain in each study were sought (e.g. for all measures, time points, analyses), and if not, the methods used to decide which results to collect.	5
	10b	List and define all other variables for which data were sought (e.g. participant and intervention characteristics, funding sources). Describe any assumptions made about any missing or unclear information.	5
Study risk of bias assessment	11	Specify the methods used to assess risk of bias in the included studies, including details of the tool(s) used, how many reviewers assessed each study and whether they worked independently, and if applicable, details of automation tools used in the process.	5-6
Effect measures	12	Specify for each outcome the effect measure(s) (e.g. risk ratio, mean difference) used in the synthesis or presentation of results.	5-6
Synthesis methods	13a	Describe the processes used to decide which studies were eligible for each synthesis (e.g. tabulating the study intervention characteristics and comparing against the planned groups for each synthesis (item #5)).	5-6
	13b	Describe any methods required to prepare the data for presentation or synthesis, such as handling of missing summary statistics, or data	5-6

Section and Topic	Item #	Checklist item	Location where item is reported
		conversions.	
	13c	Describe any methods used to tabulate or visually display results of individual studies and syntheses.	5-6
	13d	Describe any methods used to synthesize results and provide a rationale for the choice(s). If meta-analysis was performed, describe the model(s), method(s) to identify the presence and extent of statistical heterogeneity, and software package(s) used.	5-6
	13e	Describe any methods used to explore possible causes of heterogeneity among study results (e.g. subgroup analysis, meta-regression).	5-6
	13f	Describe any sensitivity analyses conducted to assess robustness of the synthesized results.	5-6
Reporting bias assessment	14	Describe any methods used to assess risk of bias due to missing results in a synthesis (arising from reporting biases).	5-6
Certainty assessment	15	Describe any methods used to assess certainty (or confidence) in the body of evidence for an outcome.	5-6
**RESULTS**			
Study selection	16a	Describe the results of the search and selection process, from the number of records identified in the search to the number of studies included in the review, ideally using a flow diagram.	7
	16b	Cite studies that might appear to meet the inclusion criteria, but which were excluded, and explain why they were excluded.	11
Study characteristics	17	Cite each included study and present its characteristics.	16
Risk of bias in studies	18	Present assessments of risk of bias for each included study.	11 Supplementary material
Results of individual studies	19	For all outcomes, present, for each study: (a) summary statistics for each group (where appropriate) and (b) an effect estimate and its precision (e.g. confidence/credible interval), ideally using structured tables or plots.	9-13
Results of syntheses	20a	For each synthesis, briefly summarise the characteristics and risk of bias among contributing studies.	9-13
	20b	Present results of all statistical syntheses conducted. If meta-analysis was done, present for each the summary estimate and its precision (e.g. confidence/credible interval) and measures of statistical heterogeneity. If comparing groups, describe the direction of the effect.	9-13
	20c	Present results of all investigations of possible causes of heterogeneity among study results.	9-13
	20d	Present results of all sensitivity analyses conducted to assess the robustness of the synthesized results.	9-13
Reporting biases	21	Present assessments of risk of bias due to missing results (arising from reporting biases) for each synthesis assessed.	11 Supplementary material
Certainty of evidence	22	Present assessments of certainty (or confidence) in the body of evidence for each outcome assessed.	Supplementary material

Section and Topic	Item #	Checklist item	Location where item is reported
**DISCUSSION**			
Discussion	23a	Provide a general interpretation of the results in the context of other evidence.	13-14
	23b	Discuss any limitations of the evidence included in the review.	13-14
	23c	Discuss any limitations of the review processes used.	13-14
	23d	Discuss implications of the results for practice, policy, and future research.	13-14
**OTHER INFORMATION**			
Registration and protocol	24a	Provide registration information for the review, including register name and registration number, or state that the review was not registered.	3
	24b	Indicate where the review protocol can be accessed, or state that a protocol was not prepared.	5
	24c	Describe and explain any amendments to information provided at registration or in the protocol.	5
Support	25	Describe sources of financial or non-financial support for the review, and the role of the funders or sponsors in the review.	1
Competing interests	26	Declare any competing interests of review authors.	1
Availability of data, code and other materials	27	Report which of the following are publicly available and where they can be found: template data collection forms; data extracted from included studies; data used for all analyses; analytic code; any other materials used in the review.	2

From: Page MJ, McKenzie JE, Bossuyt PM, Boutron I, Hoffmann TC, Mulrow CD, et al. The PRISMA 2020 statement: an updated guideline for reporting systematic reviews. BMJ 2021;372:n71. doi: 10.1136/bmj.n71

For more information, visit: http://www.prisma-statement.org/

Appendix S1 The search terms applied in a systematic search

MEDLINE (via PubMed):

(Ovarian Neoplasms OR Ovarian Neoplasms/mortality OR "ovary tumor" OR "ovary cancer" OR "ovary neoplasm" OR "ovary carcinoma" OR "ovarian cancer" OR "ovarian carcinoma" OR "ovarian neoplasms" OR "tubal cancer" OR "peritoneal cancer") AND ("poly(adp-ribose) polymerase inhibitors" OR "PARP Inhibitors" OR "PARP inhibitor" OR PARP inhibitor* OR "Poly adp-ribose" OR Olaparib OR rucaparib OR niraparib OR veliparib OR talazoparib OR pamiparib OR fluzoparib OR VEGF OR anti-VEGF OR "vascular endothelial growth factor" OR "Anti–vascular endothelial growth factor" OR "inhibitor of vascular endothelial growth factor" OR Bevacizumab OR Avastin OR cediranib OR AZ2171 OR recentin) AND random*

Cochrane Library (CENTRAL):

(Ovarian Neoplasms OR "ovary tumor" OR "ovary cancer" OR "ovary neoplasm" OR "ovary carcinoma" OR "ovarian cancer" OR "ovarian carcinoma" OR "ovarian neoplasms" OR "tubal cancer" OR "peritoneal cancer") AND ("poly(adp-ribose) polymerase inhibitors" OR "PARP Inhibitors" OR "PARP inhibitor" OR PARP inhibitor* OR "Poly adp-ribose" OR Olaparib OR rucaparib OR niraparib OR veliparib OR talazoparib OR pamiparib OR fluzoparib OR VEGF OR anti-VEGF OR "vascular endothelial growth factor" OR "Anti–vascular endothelial growth factor" OR "inhibitor of vascular endothelial growth factor" OR Bevacizumab OR Avastin OR cediranib OR AZ2171 OR recentin) AND random*

Embase:

('Ovarian Neoplasms' OR 'ovary tumor' OR 'ovary cancer' OR 'ovary neoplasm' OR 'ovary carcinoma' OR 'ovarian cancer' OR 'ovarian carcinoma' OR 'ovarian neoplasms' OR 'tubal cancer' OR 'peritoneal cancer') AND ('poly(adp-ribose) polymerase inhibitors' OR 'PARP Inhibitors' OR 'PARP inhibitor' OR 'PARP inhibitor*' OR 'Poly adp-ribose' OR 'Olaparib' OR 'rucaparib' OR 'niraparib' OR 'veliparib' OR 'talazoparib' OR 'pamiparib' OR 'fluzoparib' OR 'VEGF' OR 'anti-VEGF' OR 'vascular endothelial growth factor' OR 'Anti–vascular endothelial growth factor' OR 'inhibitor of vascular endothelial growth factor' OR 'Bevacizumab' OR 'Avastin' OR 'cediranib' OR 'AZ2171' OR 'recentin') AND random*

Figure S1 Forest plot demonstrates that the combination therapy for recurrent ovarian cancer is non-inferior to PARP inhibitor alone in reducing the hazard ratio for disease progression or death in the analyzed populations

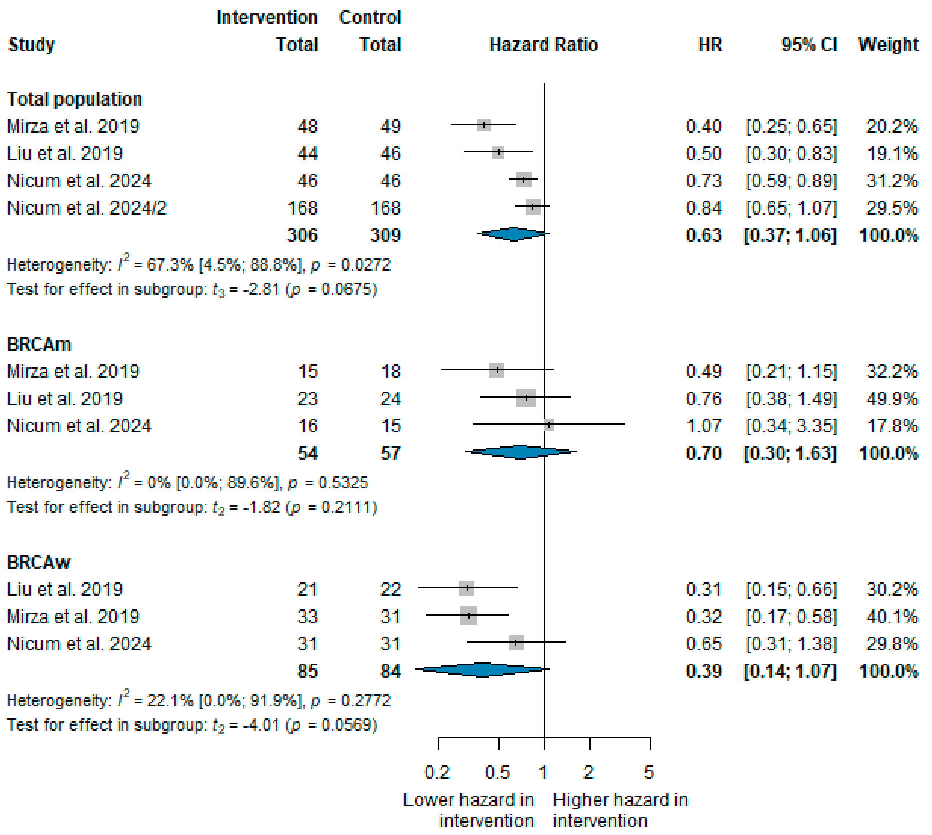

Figure S2 Forest plot demonstrates that the combination therapy for recurrent ovarian cancer is equally effective as chemotherapy alone in reducing the hazard ratio for disease progression or death in the analyzed populations

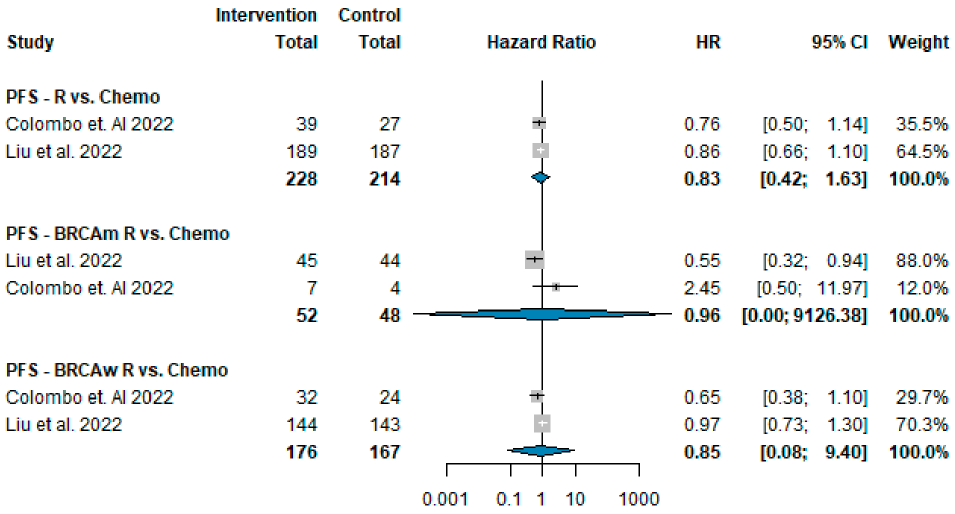

Figure S3 Forest plot demonstrates the impact of combination therapy versus PARP inhibitor alone on the risk of adverse events in patients with recurrent ovarian cancer

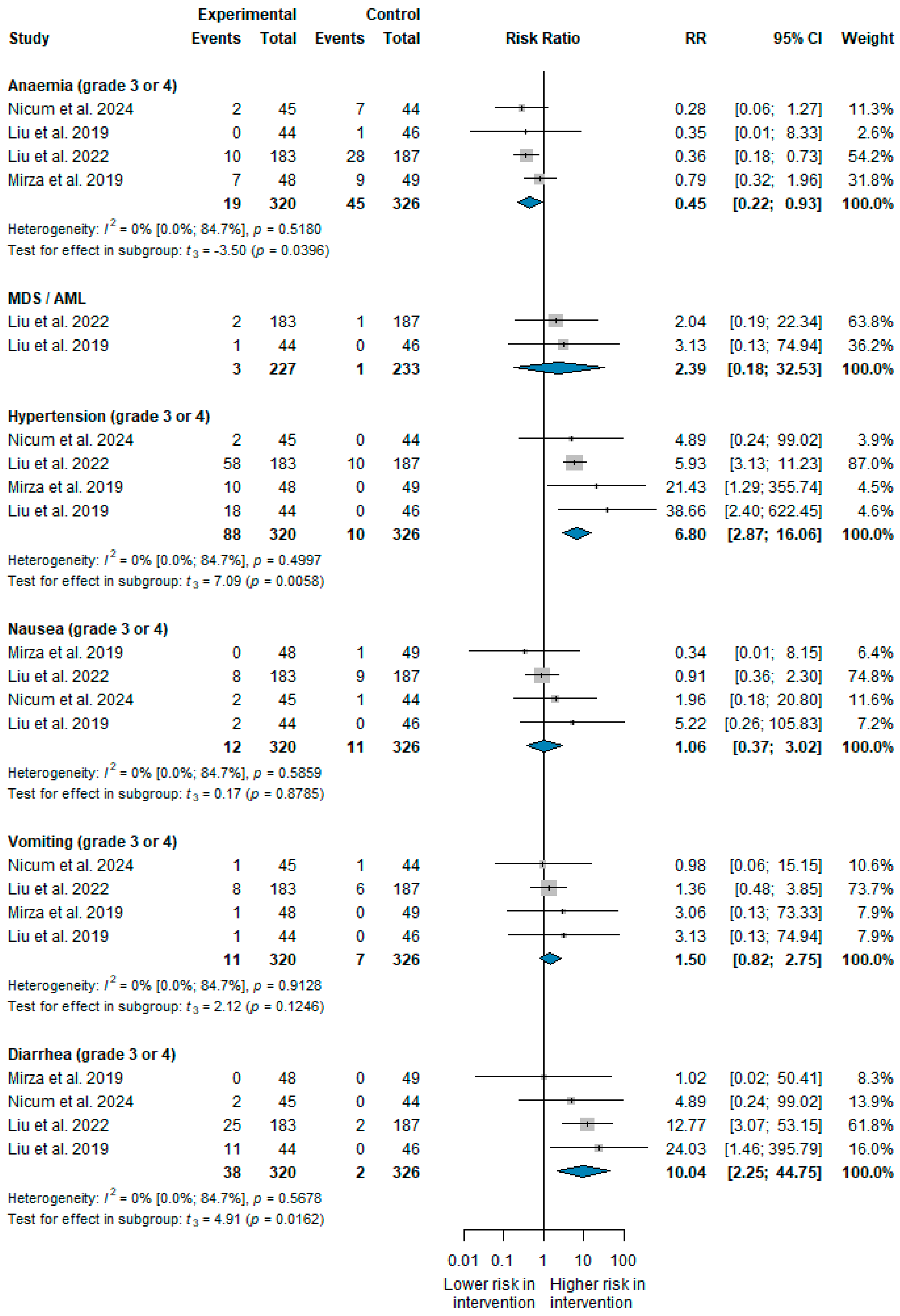

Figure S4 Forest plot demonstrates the impact of combination therapy versus chemotherapy alone on the risk of adverse events in patients with recurrent ovarian cancer

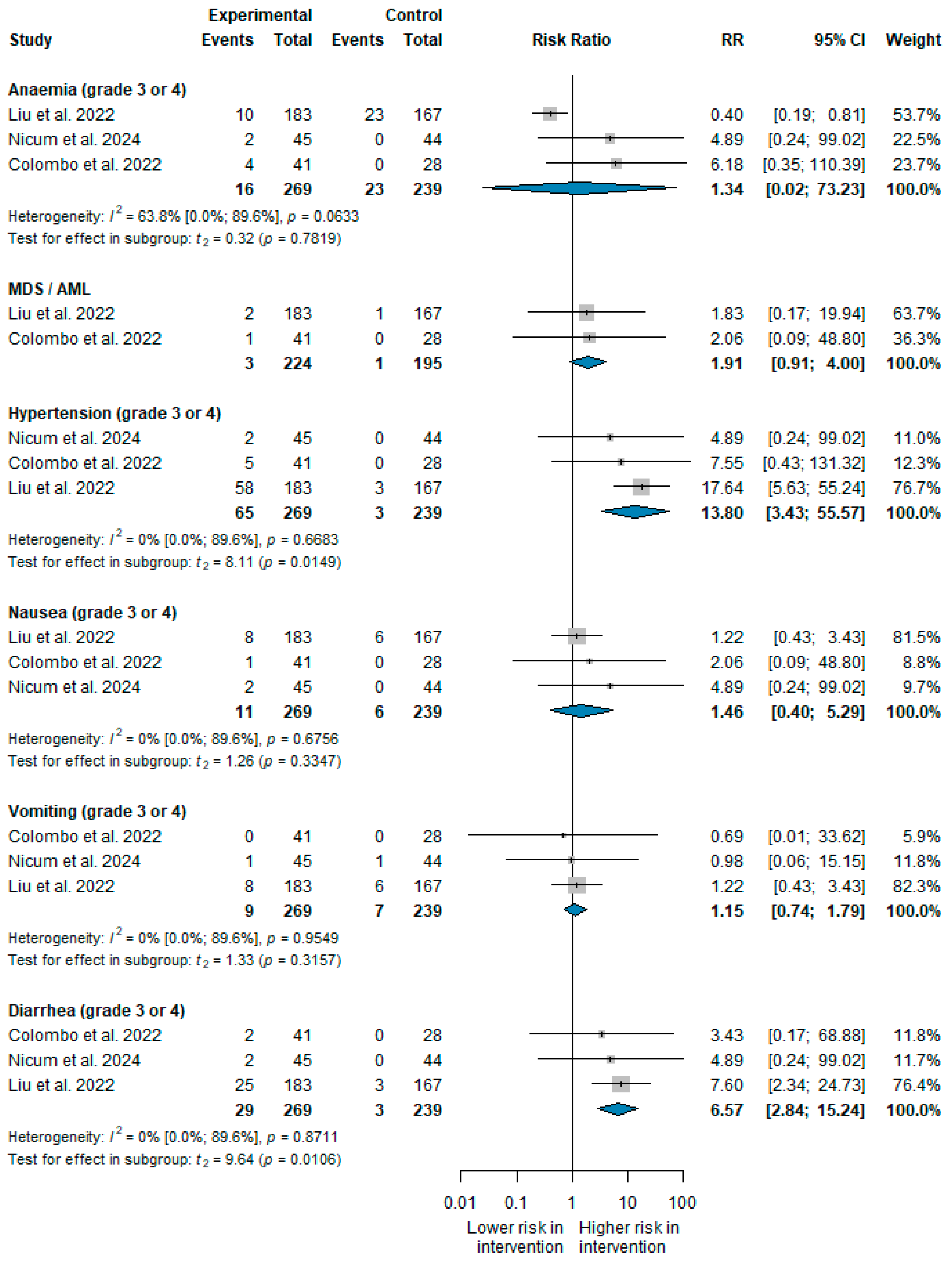

Figure S5 Risk of bias summary at study level: for each included trial

Trial	Randomization process	Deviations from intended interventions	Missing outcome data	Measurement of the outcome	Selection of the reported result	Overall
NRG-GY004	+	?	+	+	?	!
NCT01116648	+	?	+	+	+	!
AVANOVA2	+	–	+	+	?	!
BAROCCO	+	?	+	+	+	+
OCTOVA	+	+	+	+	?	+
PAOLA-1	+	+	+	+	+	+
ICON9	?	?	?	+	–	–

Table S2 Summary of findings & quality of evidence – PFS in recurrent OC: combination therapy vs. PARP inhibitor alone

Certainty assessment							Summary of findings				
Participants (studies) Follow-up	Risk of bias	Inconsistency	Indirectness	Imprecision	Publication bias	Overall certainty of evidence	Study event rates (%)		Relative effect (95% CI)	Anticipated absolute effects	
							With Combination therapy	With PARPi		Risk with Combination therapy	Risk difference with PARPi
**Total population**											
615 (4 RCTs)	not serious	not serious	serious	serious	none	⨁⨁◯◯ Low	306 participants	309 participants	**HR** (0 to 1.) [Total population]	**Low**	
										0 per 1 000	**-- per 1 000** (from -- to --)
**BRCAm**											
111 (3 RCTs)	not serious	serious	serious	serious	none	⨁⨁◯◯ Low	54 participants	57 participants	**HR 0.70** (0.30 to 1.63) [BRCAm]	**Low**	
										0 per 1 000	**-- per 1 000** (from -- to --)
**BRCAW**											
169 (3 RCTs)	not serious	not serious	serious	serious	none	⨁⨁◯◯ Low	85 participants	84 participants	**HR 0.39** (0.14 to 1.07) [BRCAW]	**Low**	
										0 per 1 000	**-- per 1 000** (from -- to --)

CI: confidence interval; **HR:** hazard Ratio; **BRCAw**: BRCA wild-type; **BRCAm**: BRCA mutated;

Table S3 Summary of findings & quality of evidence – PFS in recurrent OC: combination therapy vs. chemotherapy alone

							Summary of findings				
Participants (studies) Follow-up	Risk of bias	Inconsistency	Indirectness	Imprecision	Publication bias	Overall certainty of evidence	Study event rates (%)		Relative effect (95% CI)	Anticipated absolute effects	
							With Combination therapy	With Chemotherapy		Risk with Combination therapy	Risk difference with Chemotherapy
**Total Population**											
442 (2 RCTs)	not serious	not serious	serious	serious	none	⨁⨁◯◯ Low	228 participants	214 participants	**HR 0.83** (0.42 to 1.63) [Total Population]	Low	
										0 per 1 000	**-- per 1 000** (from -- to --)
**BRCAm**											
100 (2 RCTs)	not serious	not serious	very serious	serious	none	⨁◯◯◯ Very low	52 participants	48 participants	**HR 0.96** (0.00 to 9126.38) [BRCAm]	Low	
										0 per 1 000	**-- per 1 000** (from -- to --)
**BRCAw**											
343 (2 RCTs)	not serious	not serious	very serious	serious	none	⨁◯◯◯ Very low	176 participants	167 participants	**HR 0.85** (0.08 to 9.40) [BRCAw]	Low	
										0 per 1 000	**-- per 1 000** (from -- to --)

CI: confidence interval; **HR:** hazard Ratio; **BRCAm**: BRCA mutated; **BRCAw**: BRCA wild-type

Table S4 Summary of findings & quality of evidence – AEs in recurrent OC: combination therapy vs. PARP inhibitor alone

Certainty assessment							Summary of findings				
Participants (studies) Follow-up	Risk of bias	Inconsistency	Indirectness	Imprecision	Publication bias	Overall certainty of evidence	Study event rates (%)		Relative effect (95% CI)	Anticipated absolute effects	
							With Combination therapy	With PARPi		Risk with Combination therapy	Risk difference with PARPi
**Anaemia grade 3 & grade 4**											
646 (4 RCTs)	not serious	not serious	serious	serious	none	⨁⨁◯◯ Low	320 participants	316 participants	**RR 0.45** (0.22 to 0.93) [Anaemia grade 3 & grade 4]	**Low**	
										0 per 1 000	**0 fewer per 1 000** (from 0 fewer to 0 fewer)
**MDS / AML**											
460 (2 RCTs)	not serious	not serious	very serious	serious	none	⨁◯◯◯ Very low	227 participants	233 participants	**RR 2.41** (0.16 to 36.22) [MDS / AML]	**Low**	
										0 per 1 000	**0 fewer per 1 000** (from 0 fewer to 0 fewer)
**Nausea grade 3 & grade 4**											
646 (4 RCTs)	not serious	not serious	very serious	serious	none	⨁◯◯◯ Very low	320 participants	326 participants	**RR 1.06** (0.37 to 3.02) [Nausea grade 3 & grade 4]	**Low**	
										0 per 1 000	**0 fewer per 1 000** (from 0 fewer to 0 fewer)

Certainty assessment							Summary of findings				
**Hypertension grade 3 & grade 4**											
										**Low**	
646 (4 RCTs)	not serious	not serious	serious	not serious	none	⨁⨁⨁◯ Moderate	320 participants	316 participants	**RR 6.8** (2.87 to 16.06) [Hypertension grade 3 & grade 4]	0 per 1 000	**0 fewer per 1 000** (from 0 fewer to 0 fewer)
**Vomiting grade 3 & grade 4**											
										**Low**	
646 (4 RCTs)	not serious	not serious	very serious	serious	none	⨁◯◯◯ Very low	320 participants	326 participants	**RR 1.50** (0.82 to 2.75) [Vomiting grade 3 & grade 4]	0 per 1 000	**0 fewer per 1 000** (from 0 fewer to 0 fewer)
**Diarrhea grade 3 & grade 4**											
										**Low**	
646 (4 RCTs)	not serious	not serious	serious	not serious	none	⨁⨁⨁◯ Moderate	320 participants	326 participants	**RR 10.04** (2.25 to 44.75) [Diarrhea grade 3 & grade 4]	0 per 1 000	**0 fewer per 1 000** (from 0 fewer to 0 fewer)

CI: confidence interval; **OR:** odds ratio; **MDS / AML**: myelodysplastic syndrome or acute myeloid leukemia

Table S5 Summary of findings & quality of evidence – AEs in recurrent OC: combination therapy vs. chemotherapy alone

Certainty assessment							Summary of findings				
Participants (studies) Follow-up	Risk of bias	Inconsistency	Indirectness	Imprecision	Publication bias	Overall certainty of evidence	Study event rates (%)		Relative effect (95% CI)	Anticipated absolute effects	
							With Combination therapy	With Chemotherapy		Risk with Combination therapy	Risk difference with Chemotherapy
**Anaemia grade 3 & grade 4**											
508 (3 RCTs)	not serious	not serious	very serious	serious	none	⊕◯◯◯ Very low	269 participants	239 participants	**RR 1.34** (0.02 to 73.23) [Anaemia grade 3 & grade 4]	**Low**	
										0 per 1 000	**0 fewer per 1 000** (from 0 fewer to --)
**MDS / AML**											
419 (2 RCTs)	not serious	not serious	serious	serious	none	⊕⊕◯◯ Low	224 participants	195 participants	**RR 1.93** (0.82 to 4.54) [MDS / AML]	**Low**	
										0 per 1 000	**0 fewer per 1 000** (from 0 fewer to 0 fewer)
**Nausea grade 3 & grade 4**											
508 (3 RCTs)	not serious	not serious	very serious	serious	none	⊕◯◯◯ Very low	269 participants	239 participants	**RR 1.46** (0.40 to 5.29) [Nausea grade 3 & grade 4]	**Low**	
										0 per 1 000	**0 fewer per 1 000** (from 0 fewer to 0 fewer)

Hypertension grade 3 & grade 4

Certainty assessment							Summary of findings					
										Low	
508 (3 RCTs)	not serious	not serious	serious	serious	none	⊕⊕◯◯ Low	269 participants	239 participants	**RR 13.80** (3.43 to 55.57) [Hypertension grade 3 & grade 4]	0 per 1 000	**0 fewer per 1 000** (from 0 fewer to 0 fewer)
**Vomiting grade 3 & grade 4**											
										Low	
508 (3 RCTs)	not serious	not serious	very serious	serious	none	⊕◯◯◯ Very low	269 participants	239 participants	**RR 1.15** (0.74 to 1.79) [Vomiting grade 3 & grade 4]	0 per 1 000	**0 fewer per 1 000** (from 0 fewer to 0 fewer)
**Diarrhea grade 3 & grade 4**											
										Low	
508 (3 RCTs)	not serious	not serious	serious	not serious	none	⊕⊕⊕◯ Moderate	269 participants	239 participants	**RR 6.57** (2.84 to 15.24) [Diarrhea grade 3 & grade 4]	0 per 1 000	**0 fewer per 1 000** (from 0 fewer to 0 fewer)

CI: confidence interval; **OR:** odds ratio; **MDS / AML**: myelodysplastic syndrome or acute myeloid leukemia
